# Viral infection upregulates myostatin promoter activity in orange-spotted grouper (*Epinephelus coioides*)

**DOI:** 10.1371/journal.pone.0186506

**Published:** 2017-10-16

**Authors:** Yi-Tien Chen, Chao-Fen Lin, Young-Mao Chen, Chih-En Lo, Wan-Erh Chen, Tzong-Yueh Chen

**Affiliations:** 1 Laboratory of Molecular Genetics, Department of Biotechnology and Bioindustry Sciences, College of Bioscience and Biotechnology, National Cheng Kung University, Tainan, Taiwan; 2 Laboratory of Molecular Genetics, Institute of Biotechnology, College of Bioscience and Biotechnology, National Cheng Kung University, Tainan, Taiwan; 3 Translational Center for Marine Biotechnology, National Cheng Kung University, Tainan, Taiwan; 4 Agriculture Biotechnology Research Center, National Cheng Kung University, Tainan, Taiwan; University of Hong Kong, HONG KONG

## Abstract

Myostatin is a negative regulator of myogenesis and has been suggested to be an important factor in the development of muscle wasting during viral infection. The objective of this study was to characterize the main regulatory element of the grouper myostatin promoter and to study changes in promoter activity due to viral stimulation. *In vitro* and *in vivo* experiments indicated that the E-box E6 is a positive *cis*-and *trans*-regulation motif, and an essential binding site for MyoD. In contrast, the E-box E5 is a dominant negative *cis-*regulatory. The characteristics of grouper myostatin promoter are similar in regulation of muscle growth to that of other species, but mainly through specific regulatory elements. According to these results, we conducted a study to investigate the effect of viral infection on myostatin promoter activity and its regulation. The nervous necrosis virus (NNV) treatment significantly induced myostatin promoter activity. The present study is the first report describing that specific myostatin motifs regulate promoter activity and response to viral infection.

## Introduction

Myostatin (GDF-8) is a member of the transforming growth factor-β (TGF-β) superfamily and acts as a negative regulator of skeletal muscle growth and development [1–4]. The characterization and expression of the myostatin gene has been studied in several fish species, such as channel catfish (*Ictalurus punctatus*), yellow catfish (*Pelteobagrus fulvidraco*), zebrafish (*Danio rerio*), gilthead seabream (*Sparus aurata*), orange-spotted grouper (*Epinephelus coioides*), Atlantic salmon (*Salmo salar*), rainbow trout (*Oncorhynchus mykiss*), Mozambique tilapia (*Oreochromis mossambicus*), white bass (*Morone chrysops*), striped bass (*Morone saxatilis*) and white perch (*Morone americana*) [5–14]. We previously reported that the full orange-spotted grouper myostatin mRNA length is 2776 bp with three exons. Its promoter includes 1974 bp located 5’ upstream of the initiation site of myostatin mRNA, and contains ten E-box motifs and multiple muscle regulatory response elements, such as muscle-specific Mt binding site (MTBF), and the growth factor independence 1 zinc finger protein (Gfi-1B) [5]. Although E-box motifs and muscle growth regulatory elements at myostatin promoter have been found in mammals, such as bovine (*Bos taurus*) and humans (*Homo sapiens*), the number and arrangement of motifs and elements in mammals vary and are species dependent [15, 16]. Therefore, the regulation of myostatin promoter could be very different between fish and other species.

The E-Box is one of major regulatory motifs on the muscle-specific gene expression [17–19]. Myostatin contains several E-boxes at its promoter and commonly includes one specific E-box which has a major effect on the activity of the promoter [16]. This specific E-box varies in location depending on the species; in the mouse (*Mus musculus*) it is the fifth E-box, in bovine it is the sixth E-box, and in sheep (*Ovis aries*) it is the seventh E-box [16, 20, 21]. The E-box is a binding site for myogenic regulatory factors (MRFs) that include MyoD, Myogenic factor 5 (Myf-5), MRF4, and E47 [16, 22–24]. MyoD and other MRFs activate the muscle-specific gene transcription *in vitro* through binding the specific sequence CANNTG (E-box) at the promoter of muscle-specific gene [25, 26].

In addition to regulating muscle growth and development, myostatin is involved in immune response of viral infection. Our previous study indicated that grouper myostatin protein expression in serum was altered in groupers infected with nodavirus [5]. Previous study reported that porcine reproductive and respiratory syndrome virus (PRRSV)-induced pneumonia significantly upregulated myostatin mRNA expression and led to decreased muscle weight, decreased weight gain, and decreased protein accretion in adult pigs [27]. In addition, red spotted grouper nervous necrosis virus (RGNNV) infection did not trigger interferon-stimulated genes (ISGs) expression in myostatin-mutated medaka [28]. ISGs is able to activate and modify interferon-induced double-stranded RNA-dependent protein kinase which acts as an important component in the fish innate immune system to against double-stranded RNA virus infection through inhibition of protein synthesis [29–31]. In muscle wasting of cancer cachexia, activation of nuclear factor-κB (NF-κB), and subsequent depression of protein synthesis and increased muscle protein degradation [32, 33]. Moreover, NF-κB activation by tumor necrosis factor-α leads to reduction of MyoD mRNA expression [34].

However, the effect of viral infection on the transcriptional regulation of myostatin remains unclear. The objective of this study was to characterize transcriptional regulation of grouper myostatin and further study the role of myostatin in immune response to viral infection.

## Results

### Identification of *cis*-elements essential for the regulation of grouper myostatin promoter *in vitro* and *in vivo*

**Fig 1:**
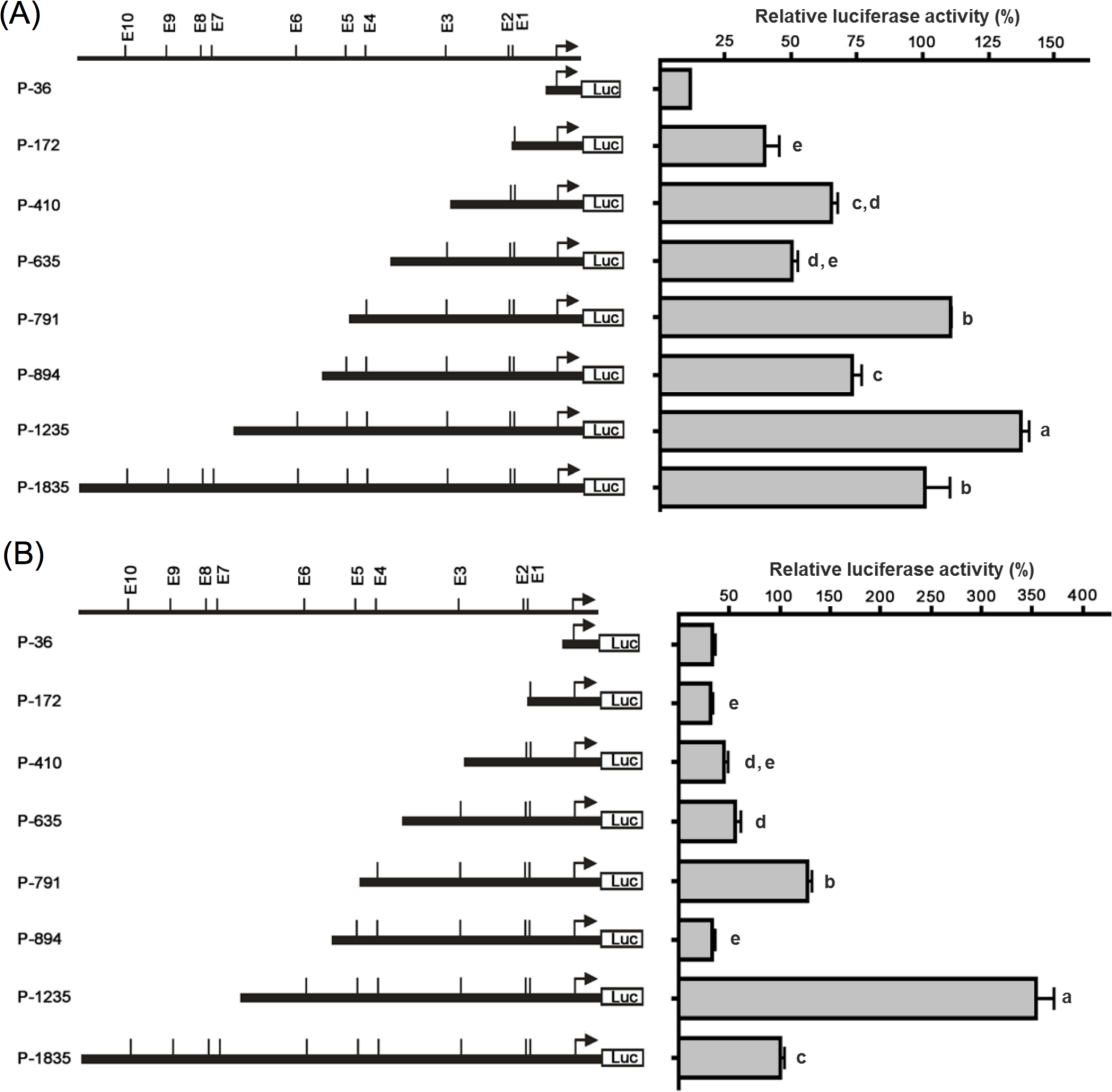
Deletion analysis of grouper myostatin 5’-flanking regulatory regions *in vitro* and *in vivo*. The seven promoter constructs were cloned into the luciferase reporter vector pGL3-Basic (left) and transfected into GF-1 cells (A) and grouper larvae muscle (B). The locations of the E-boxes of the grouper myostatin promoter are presented on the top left. The relative luciferase activity was normalized against the activity of the pGL3-Basic vector, and the results are shown in the right panel. Bars indicate averages ± standard deviations for three replicates and asterisks show significant differences (***p < 0.001).

The systematic 5’-deletion constructs of myostatin promoter was performed to define the critical E-boxes that are essential for the *cis*-transcriptional activity of the myostatin *in vitro* and *in vivo*. The seven promoter fragments containing different numbers of E-boxes were cloned into pGL3-Basic vector which carries the luciferase gene and transfected into GF-1 cells and grouper larvae muscle tissue to identify the major *cis*-acting E-box (Fig 1). The fragments deleted from the 5’-end of myostatin promoter were P-1235, P-894, P-791, P-635, P-410, P-172 and P-36 constructs which contained the region from 5’-end of E-box E1-E6, E1-E5, E1-E4, E1-E3, E1-E2, E1 and vector only, respectively. The P-1835 construct, which contained the full-length promoter, served as control. Comparison of the promoter activity *in vitro* (Fig 1A) and *in vivo* (Fig 1B) of constructs of P-894 (E-box E1-E5) and P-791 (E-box E1-E4) showed that the promoter activity significantly decreased in the presence of E-box E5 (Fig 1). The activity of the promoter was restored in the presence of E-box E6 (P-1235) and the increase was significantly higher than (P-1835) both *in vitro* and *in vivo* (Fig 1).

### Regulation of E-Box E6 and E5 on grouper myostatin promoter activity

**Fig 2:**
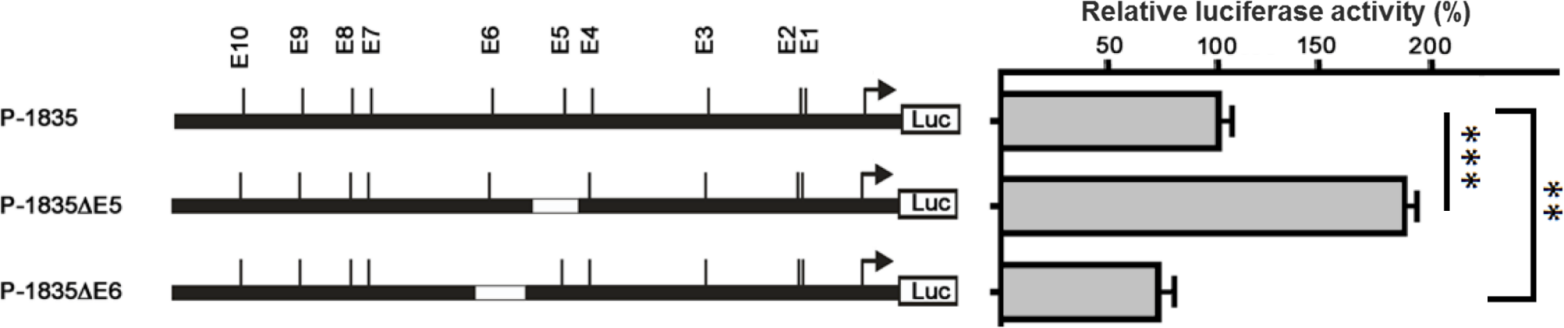
E-box E5 and E6 regulation of grouper myostatin promoter activity. The construct containing internal truncations of E-box E5 (P-1835ΔE5) or E6 (P-1835ΔE6) was transfected into GF-1 cells, respectively, and luciferase reporter activity was measured. The relative luciferase activity was normalized against that of the pGL3-Basic vector. Bars indicate averages ± standard deviations for three replicates and asterisks show significant differences (**p < 0.01, ***p< 0.001).

Because the E-box E6 and E5 region showed opposite effects on myostatin activity, we truncated the E-box E5 (P-1835ΔE5) and E6 (P-1835ΔE6) to further study the *cis-*regulation of individual E-box. Truncation of E-box E5 (P-1835ΔE5) resulted in an increase in promoter activity that was about significant higher than control (P-1835) (Fig 2). In contrast, truncation of E-box E6 resulted in a significant reduction of promoter activity (Fig 2).

### E-box specific regulation of MyoD binding

**Fig 3:**
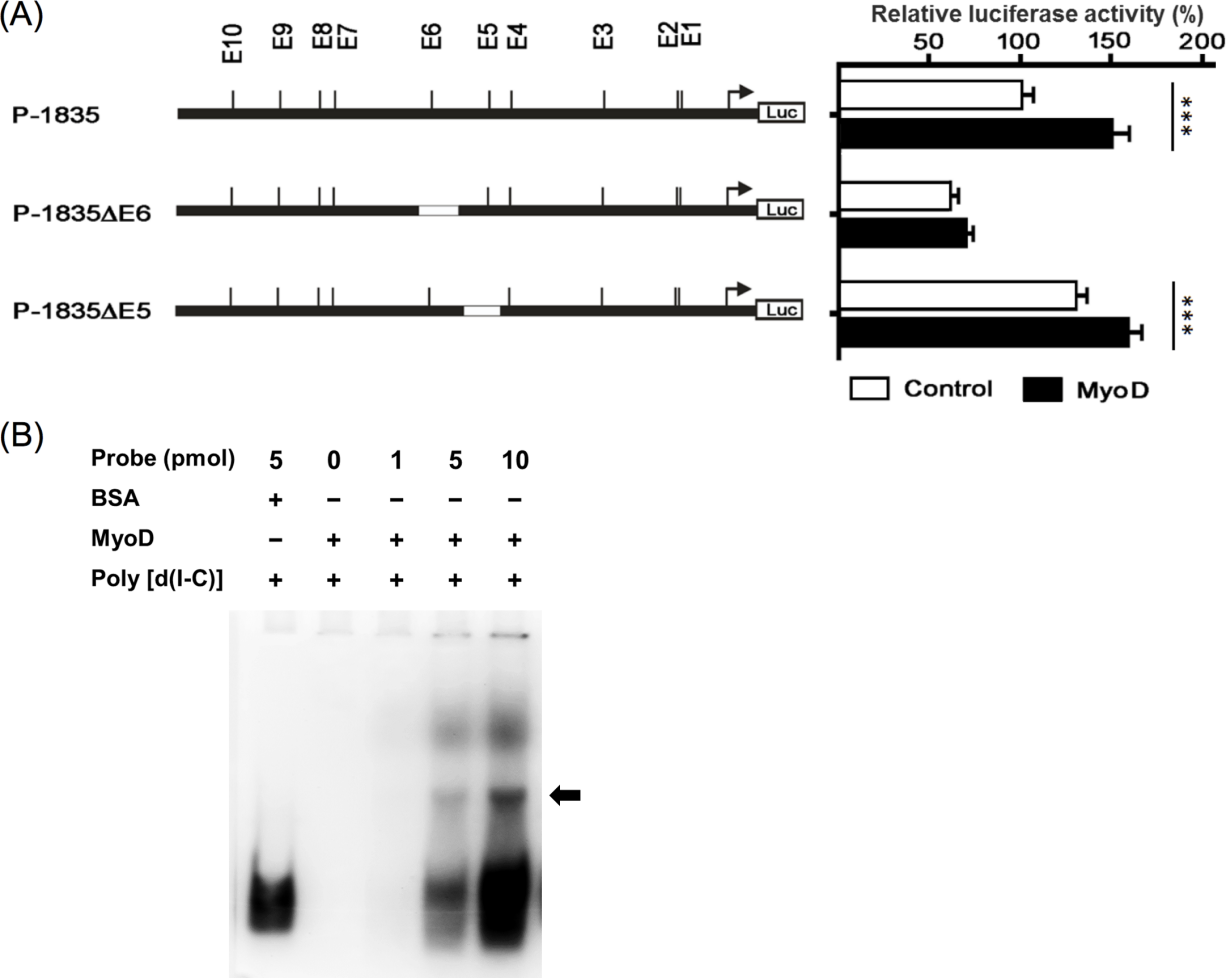
The *trans-*regulation of MyoD through the E-boxes. (A) Orange-spotted grouper MyoD gene were cloned in the expression vector pcDNA3.1 and cotransfected with the construct containing internal truncations of E-box E5 (P-1835ΔE5) or E6 (P-1835ΔE6). Bars indicate averages ± standard deviations for three replicates and asterisks show significant differences (***p < 0.001). (B) The electrophoretic mobility shift assay of E-box E6 and MyoD. Cy3-labeled DNA probes were incubated with BSA or purified recombinant MyoD (2 μg). The arrow indicates the resulting protein-DNA complexes separated from free DNA probe.

The truncated E-box E5 or E6 myostatin promoter constructs and a MyoD containing plasmid were cotransfected into GF-1 cells to investigate the *trans-*regulation of myostatin promoter by MyoD. Expression of MyoD significantly enhanced myostatin promoter activity with both the E-box E5 truncation (P-1835ΔE5) and the control (P-1835) plasmids (Fig 3A). The truncation of E-box E6 (P-1835ΔE6) led to a reduction in myostatin promoter activity, and this reduction was not compensated by MyoD expression (Fig 3A). Using E-box E6 region (30 nucleotides) as a probe in electrophoretic mobility shift assay (EMSA), the recombinant MyoD bound to the probes along with the increase of probe concentration (Fig 3B).

**Fig 4:**
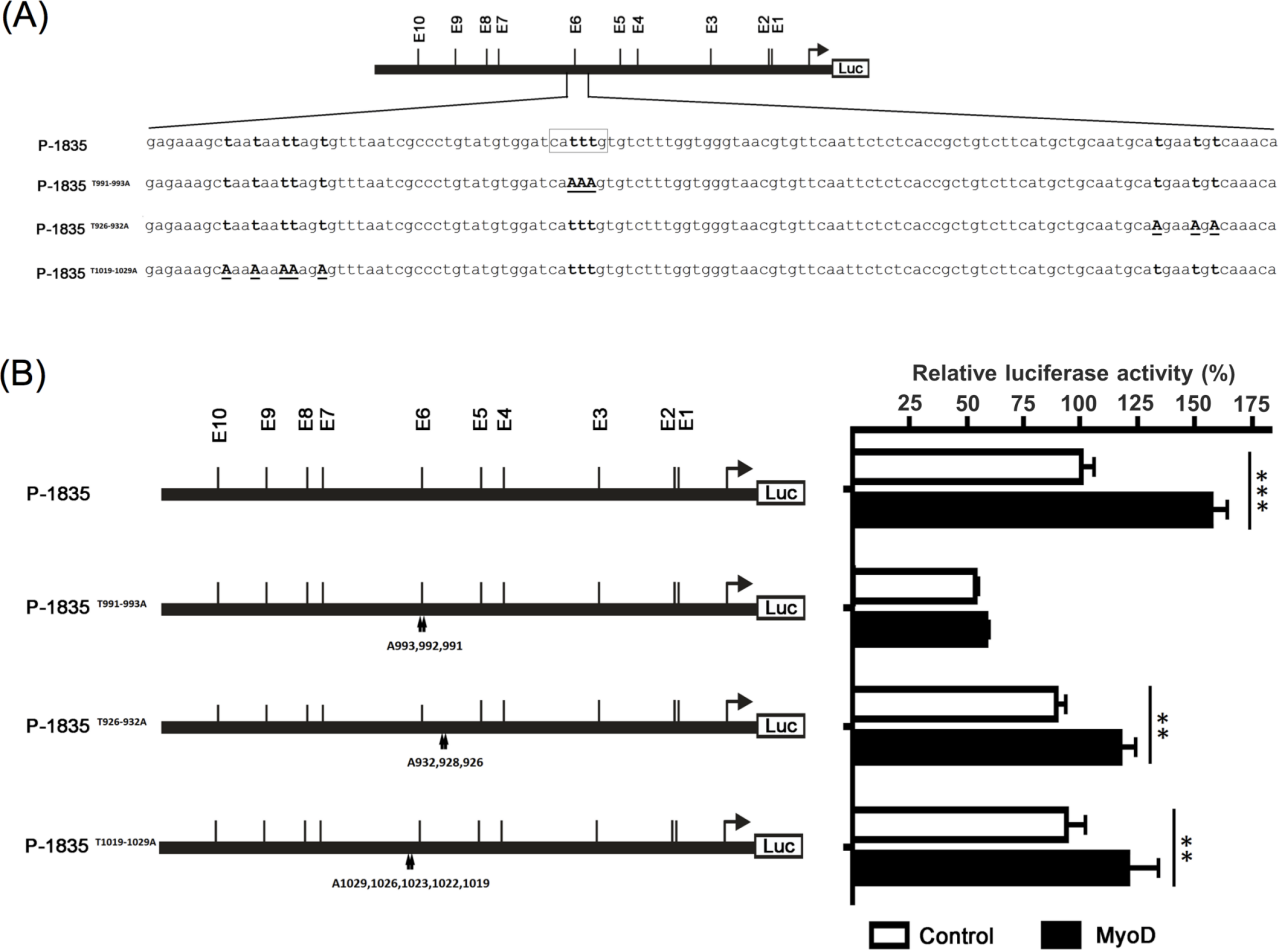
Characterization of the MyoD binding site. (A) The mutation site of E-box E6 (P-1835^T991-993A^), CdxA (P-1835^T926-932A^) and HNF-3b (P-1835^T1019-1029A^) elements were constructed by PCR-directed mutagenesis. The boxed region indicated the putative E-box E6 and the mutated nucleotides presented in underlined and in capital letters. (B) The promoter activity of E-box E6 (P-1835^T991-993A^), CdxA (P-1835^T926-932A^) and HNF-3b (P-1835^T1019-1029A^) mutated constructs were analyzed. Bars indicate averages ± standard deviations for three replicates and asterisks show significant differences (**p < 0.01, ***p < 0.001).

Bioinformatics analysis using TESS showed that the truncated E-box E6 region (-1235~-895) contained three elements, which included E-box E6, CdxA and HNF-3b regulatory sites. Therefore, the three myostatin promoter constructs with point mutation in E-box E6 (P-1835^T991-993A^), CdxA (P-1835^T926-932A^) and HNF-3b (P-1835^T1019-1029A^) were cotransfected with a MyoD expressing plasmid (Fig 4A). Unlike other constructs, the promoter activity of the E-box E6 mutant construct (P-1835^T991-993A^) was significantly decreased and could not be upregulated by MyoD expression (Fig 4B).

### Effect of NNV infection on the regulation of grouper myostatin promoter activity

**Fig 5:**
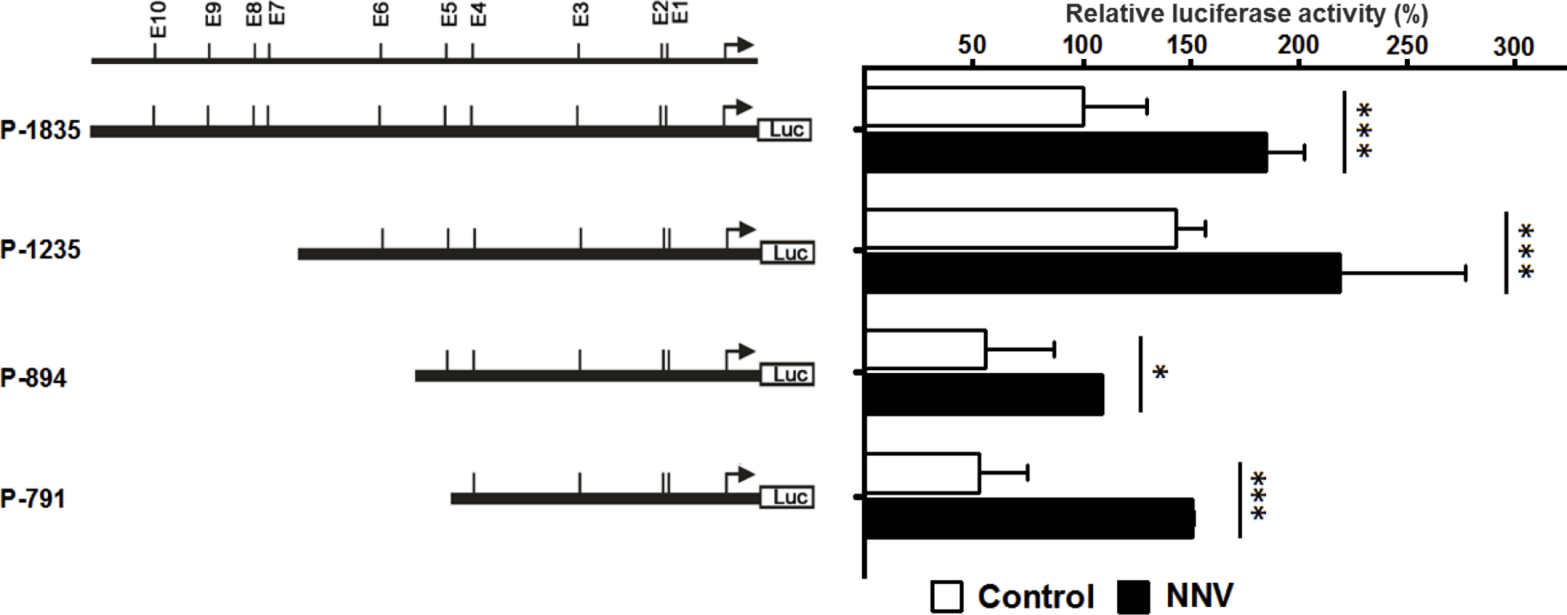
Effect of NNV infection on the myostatin promoter activity. The transfected Gf-1 cells were infected by a titter of 10^4^ TCID_50_/ml of isolated GNNV. The relative luciferase activity of constructs P-1835, P-1235, P-894 and P-791 were measured at 24 hours post-infection and normalized against the activity of the pGL3-Basic vector. Bars indicate averages ± standard deviations for three replicate experiments and asterisks show significant differences (*p < 0.05).

The infection of a single-stranded RNA virus, NNV, significantly upregulated the myostatin promoter activities of the full-length (P-1835) and different deletion promoter constructs (P-1235, P-894 and P-791) (Fig 5). These results indicate the immune response of single-stranded RNA virus infection upregulated myostatin promoter activity.

## Discussion

Myostatin inhibits muscle cell growth and differentiation and this inhibition has been reported to cause muscle hyperplasia and hypertrophy [4]. However to date, the transcriptional regulation of grouper myostatin gene has not been investigated. The present study found, using deletion analysis, that E-box E6 was the most important positive *cis*-element; deletion analysis of E-box E6 resulted in a significant reduction in promoter activity. The results of both deletion and truncation analyses indicated that the E-box E6 region performed a dominant positive effect upon the activation of orange-spotted grouper myostatin promoter.

Unlike the positive regulation of E-box E6, both the 5’-deletion and truncation analyses indicated that grouper myostatin promoter activity was significantly increased in the absence of E-box E5. Several negative transcriptional regions of myostatin promoter in other species have been reported. Deng et al. reported that the deletion of -1519 to -1236 bp of 5’-upstream of the pig myostatin promoter increased the promoter transcriptional activity in myoblast cells [35]. The -1716 to -1850 bp of 5’-upstream of *Sparus aurata* myostatin 2 promoter was identified as a negative regulatory region [36]. However, none of these studies identified a specific negative motif at the myostatin promoter. In this study, we identified E-box E5 as an important negative *cis*-element that acts to downregulate myostatin promoter activity.

MyoD is a myogenesis regulator that regulates muscle differentiation and proliferation through binding the E-boxes on the target genes [17, 19]. The pig myostatin promoter activity was increased 5-fold in C_2_C_12_ cells by cotransfection with MyoD [35]. Similarly, the grouper myostatin promoter was significantly activated by MyoD cotransfection, but not in the absence of E-box E6. These findings suggest that the grouper E-box E6 is a MyoD binding site and that MyoD binding upregulates grouper myostatin promoter activity. Since E-box E5 truncation shows no effect on the MyoD-myostatin promoter interaction, the E-box E5 may not be essential for MyoD to activate the myostatin promoter. In addition, interaction between E-box E6 and MyoD was proven through EMSA using recombinant MyoD and the oiligonucleotide fragment corresponding to the E-box E6 region. The direct binding between the E-box E6 region and MyoD increased with increase in the amount of the probe. However, E-box E6 truncated region contains not only E-box E6 but also CdxA and HNF-3b. With the use of point mutation, the E-Box E6 was further identified as a crucial motif required for MyoD activation of the grouper myostatin promoter.

Based on the results of promoter characterization, the grouper myostatin showed similar characteristics in muscle growth to that of other species. The regulation of grouper myostatin promoter is mainly through the specific regulatory elements. These results are important foundations for further investigation of the effect of viral infection on myostatin promoter activity and its regulation. Myostatin is proposed to be a regulator that involves the immune response, because we previously reported that amount of myostatin in grouper serum was affected by viral infection [5]. The muscle wasting and protein synthesis reduction occurring along with viral inflammation makes myostatin a potential factor of immune response [37]. However, the role of myostatin in immune response is still unknown. The alterations of muscle mass seen in viral infection are associated with the expression of myostatin [38]. Myostatin upregulation has been reported in different diseases with muscle wasting, such as HIV and cancer [39, 40]. At present, very little is known regarding the regulation and expression of fish myostatin in viral infected fish, especially its role in fish immune response. Thus, we applied NNV to study the effect of single-stranded RNA virus on the myostatin regulation. The significant induction of grouper myostatin promoter activity by NNV infection suggested that myostatin plays an important role in immune activity.

Decreased appetite and weight loss are common clinical signs of viral infected animals, including fish. The feed intake and growth rate of Atlantic salmon are greatly reduced by infectious pancreatic necrosis virus (IPNV) [41]. Nutritional change also affects muscle mass through the regulators of muscle growth and development. Several *in vitro* studies indicate that starving and lack of essential nutrients for muscle growth stimulate myostatin expression in fish [42–44]. Therefore, using *in vitro* experiments it is difficult to determine whether myostatin upregulation in viral infected animals is triggered by immune response or lack of nutrients due to loss of appetite. Our *in vivo* results exclude the effect of appetite and nutrient level, and indicate that viral infection regulates the muscle growth and development at the transcriptional level.

**Fig 6:**
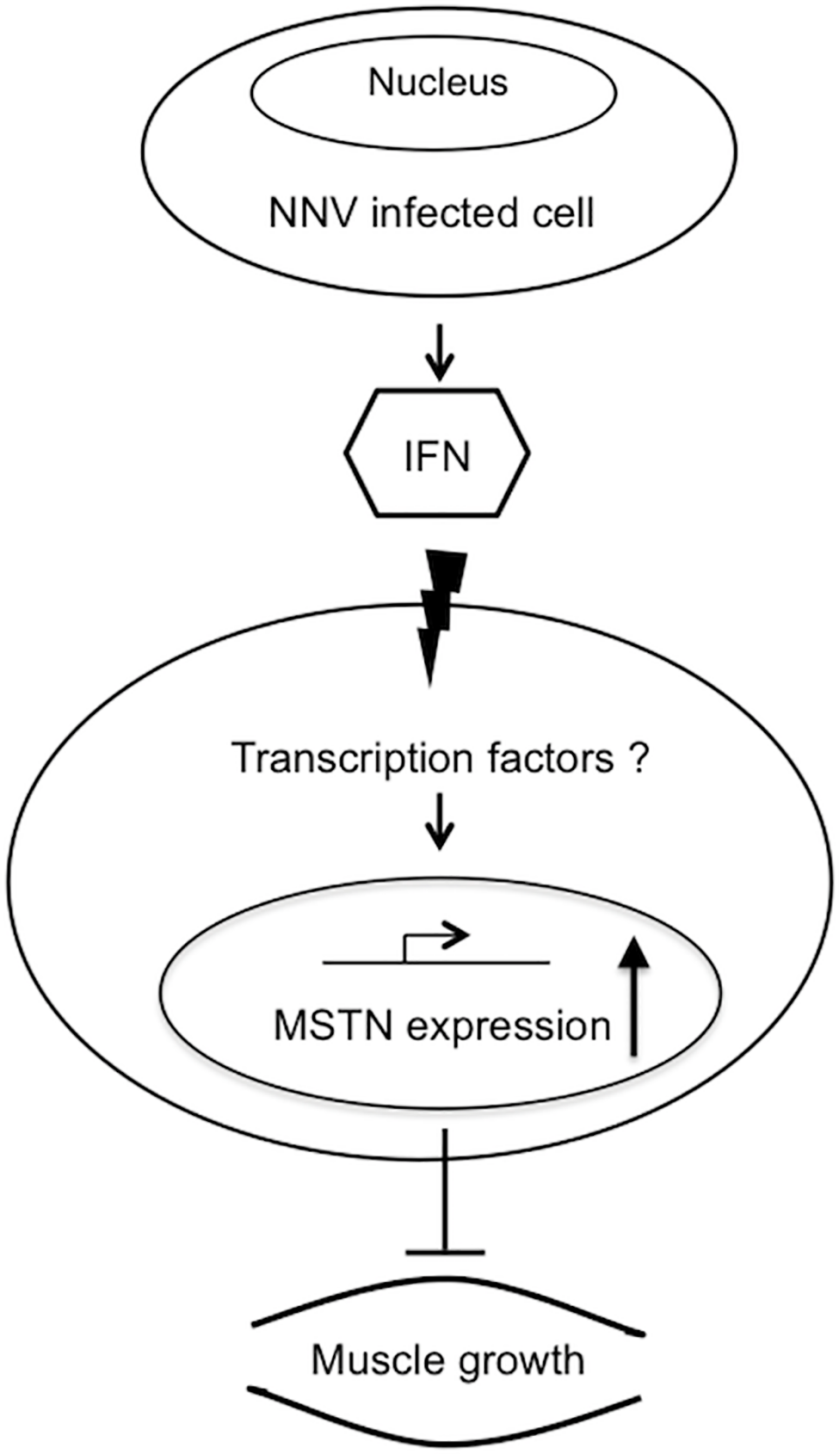
Simplified representation of the immune response to viral infection downregulates muscle growth. Infection of nervous necrosis virus (NNV) triggered antiviral response, which activated the interferon (IFN) pathway. The immune response of NNV infection upregulated myostatin (MSTN) promoter activity.

This study provides the novel findings of transcriptional regulation between viral infection and activation of myostatin promoter. E-box E6 and E5 are positive and negative *cis*-transcriptional elements, respectively, and important motifs for immune response (Fig 6). However, further investigation is needed to clarify the regulation of myostatin promoter activity during viral infection.

## Materials and methods

### Ethics statement

The research were carried out in accordance with the guidelines from Institute Animal Care and Use Committee (IACUC) of National Cheng Kung University which includes Animal Research: Reporting of In Vivo Experiments (ARRIVE) guidelines as part of it. The protocol of animal experiments was reviewed and approved by the IACUC of National Cheng Kung University (IACUC#100065).

### Bacteria strain, cell culture, fish maintenance, chemicals and bioinformatics tool

*Escherichia coli* HB101 (Promega, USA) and BL21(DE3) (Yeastern Biotech, Taiwan) cells were grown in LB medium, with or without appropriate antibiotics, and incubated at 37°C with shaking. Grouper fin cells (GF-1, BCRC 960094) [45] were obtained from Bioresource Collection and Research Center (BCRC) in Taiwan. GF-1 cells were maintained in antibiotic-free Leibovitz’s L-15 medium (GIBCO, USA) with 5% v/v heat-inactivated fetal bovine serum (FBS, GIBCO, USA) at 28°C. Grouper larvae, obtained from Translational Center for Marine Biotechnology of National Cheng Kung University (Tainan, Taiwan), were fed a combination of live brine shrimp and its nauplii in a tank filled with air-pumped circulating seawater at 28°C. The working concentration of ampicillin (Sigma-Aldrich, USA) and kanamycin (Sigma-Aldrich, USA) in this study was 100 μg/mL and 50 μg/mL, respectively. The sequence of the grouper myostatin promoter was analyzed with the transcription element search system (TESS) program (Baylor College of Medicine Human Genome Sequencing Center) for transcriptional elements.

### Construction of myostatin promoter-luciferase plasmid

A full-length 1835-bp fragment of orange-spotted grouper myostatin promoter was cloned into pGEM-T Easy vector (Promega, USA) [5]. The serial 5’-end deleted, internal truncated, and point mutated promoters were inserted into the reporter plasmid pGL3-Basic (Promega, USA) which contains the luciferase reporter gene. Plasmids were constructed by standard PCR methods using 1 unit *Pfu* DNA polymerase (Promega, USA) with the primers listed in Table 1. PCR was performed using the following protocol: initial denaturation for 3 min at 94°C; followed by 30 cycles of denaturation for 30 sec at 94°C, annealing for 15 sec at 50°C, and extension for 2 min at 72°C; then the final extension was for 5 min at 72°C. All PCR products were digested by Nhe I and Hind III and gel-purified using QIAquick Gel Extraction Kit (Qiagen, Germany). The 5’-deletion fragments of the myostatin promoter containing different numbers of E-box motifs were cloned into the pGL3-Basic vector digested by NheI and Hind III. Full-length cDNA encoding the orange-spotted grouper MyoD was amplified by PCR with primer pairs MyoDTF and MyoDTR (Table 1) and cloned into the pcDNA^TM^ 3.1 vector (Invitrogen, USA) to obtain the plasmid pcDNA-myoD.

**Table 1:** Primers used in this study.

Name	Primer sequences (5’–3’)	Position*	Application
PMF	CTAGCTAGCTCTTAATAATAAGCAAT	-1835	To construct myostatin promoter reporter plasmids
PMR	CCCAAGCTTTGTCTCTAAAGTGTGCAG	-1	To construct myostatin promoter reporter plasmids
PE6F	ACAGCTAGCATATTTATGCCCCTATAATG	-1235	To construct 5’-deletion of myostatin promoter reporter plasmid
PE5F	AGTGCTAGCTGAAAGTACCTGGAGAAT	-894	To construct 5’-deletion of myostatin promoter reporter plasmid
PE4F	TGAGCTAGCGAGATGATGGCATTTCTG	-791	To construct 5’-deletion of myostatin promoter reporter plasmid
PE3F	ATCGCTAGCAGTTTTGACTTAACATG	-635	To construct 5’-deletion of myostatin promoter reporter plasmid
PE2F	TGAGCTAGCGGTGTATTCTTTTGGAG	-410	To construct 5’-deletion of myostatin promoter reporter plasmid
PE1F	CTGGCTAGCCGTTGAGCACATGCTCAC	-172	To construct 5’-deletion of myostatin promoter reporter plasmid
PTATAF	CTAGCTAGCGAGTATAAAAAGGTGTG	-36	To construct 5’-deletion of myostatin promoter reporter plasmid
PΔE6F	GAGTTGTCCAGAGAATCTGAAAGTACCTGG	-1033	To construct E box 6 truncation of myostatin promoter reporter plasmid
PΔE6R	CCAGGTACTTTCAGATTCTCTGGACAACTC	-897	To construct E box 6 truncation of myostatin promoter reporter plasmid
PΔE5F	GAAAGTACCTGGAGAATGCCACACGATGGC	-877	To construct E box 5 truncation of myostatin promoter reporter plasmid
PΔE5R	GCCATCGTGTGGCATTCTCCAGGTACTTTC	-754	To construct E box 5 truncation of myostatin promoter reporter plasmid
T926AF	CTGCAATGCAAGAAAGACAAACACTTAAAA	-926	To construct E6 point mutation of myostatin promoter reporter plasmid
T926AR	TTTTAAGTGTTTGTCTTTCTTGCATTGCAG	-926	To construct E6 point mutation of myostatin promoter reporter plasmid
T991AF	TATGTGGATCAAAAGTGTCTTTGGT	-991	To construct E6 point mutation of myostatin promoter reporter plasmid
T991AR	ACCAAAGACACTTTTGATCCACATA	-991	To construct E6 point mutation of myostatin promoter reporter plasmid
T1019AF	CCAGAGAAAGCAAAAAAAAAGAGTTTAATC	-1019	To construct E6 point mutation of myostatin promoter reporter plasmid
T1019AR	GATTAAACTCTTTTTTTTTGCTTTCTCTGG	-1019	To construct E6 point mutation of myostatin promoter reporter plasmid
MyoDTF	CACGCTAGCCGCCATGGATCTCTCCGACCTTC		To construct myoD plasmid, pcDNA-myoD for transfection
MyoDTR	CCTCTCGAGTCAGAGCGGCTCGAAGATGC		To construct myoD plasmid, pcDNA-myoD for transfection
MyoDF	CTTCATATGGATCTCTCCGACCTTCCC		To construct myoD expression plasmid for *E*. *coli*
MyoDR	TCACTCGAGGAGCGGCTCGAAGATG		To construct myoD expression plasmid for *E*. *coli*
E6F	CTGTATGTGGATCATTTGTGTCTTTGGTGG		Cy3 labelled and used for EMSA assay
E6R	CCACCAAAGACACAAATGATCCACATACAG		Cy3 labelled and used for EMSA assay

Restriction enzyme recognition sites introduced in some oligonucleotides are underlined.

*The position indicates the primer binding site on the myostatin promoter.

### Transfection of deletion analysis

One day before transfection, GF-1 cells were seeded into 6-well plates (0.3x10^6^ cells/well) and grown to 85–90% confluence. Transfections were carried out using the Lipofectamine^TM^ 2000 (Invitrogen, USA) according to the manufacturer’s instructions. Prior to transfection, cells were washed twice with PBS (pH 7.4) and the cell medium was replaced by serum-free L-15 medium. For each well, 2 μg of total plasmid DNA (using 1 μg each of plasmid DNAs while performing the cotransfection with pcDNA-myoD) and 10 μL lipofectamines were first diluted respectively with 250 μL serum-free L-15 medium and incubated for 5 min at room temperature, and subsequently the diluted plasmids combined with diluted lipofectamines were mixed gently and incubated for 30 min at room temperature before the mixtures were applied to the cells. A plasmid carrying a renilla luciferase gene was cotransfected with the reporter construct and was used for normalization of transfection efficiency. The cells were cultured for 4 h and then the transfection medium was replaced by fresh L-15 medium containing 5% FBS. Cells were cultured for further 48 h, and subsequently assayed for luciferase activity.

### DNA injection and electroporation in grouper larvae

The method of fish experiments approved by the IACUC of National Cheng Kung University (IACUC#100065). The grouper larvae (average body length of 2.5 cm and average body weight of 1 g) were used for *in vivo* experiments. Tweezer type electrodes (Super Electroporator NEPA21, NEPA GENE CO., LTD, Japan) and syringe needles 30 gauge were used to electroporate DNA into grouper muscle. The fish were temporarily removed from the water and restrained on a soft wedge and given an intra-muscular injection of 20 μg plasmid DNA in PBS near the dorsal fin. Immediately after injection, electrical pulses were applied to the fish by holding it between the electrodes. The pulses consisted of two trains of 4 pulses (100V/cm, 50 msecs positive and 950 msecs negative). The fish were immediately released back into the tanks. The euthanasia of fish were approved by the IACUC of National Cheng Kung University and carried out in accordance with the Appendix section of Review of Schedule 1 of the Animals (Scientific Procedures) Act 1986. After 48 hour, the fish were euthanized by immersing in a seawater tank with 300 mg/L MS-222 buffered to pH 7.0–7.5 and remained in the solution 15 minutes following cessation of opercula movement [46]. The muscle cells were collected for luciferase assays.

### Luciferase assays

Luciferase activities were measured using the Dual-Luciferase^®^ Reporter Assay System (Promega, USA). Cells were cultured in the 6-well plates and harvested 48 h after addition of DNA-Lipofectamine complexes. The growth medium was removed and the cells were washed twice with PBS, covered with 500 μL/well passive lysis buffer (Promega, USA) for 15 min, followed by collection of cells and centrifugation to remove the debris. Subsequently, 20 μL of cell lysates from each well was transferred into the luminometer tube containing 100 μL Luciferase Assay Reagent II. The firefly luciferase activity of cell lysates was measured by luminometer (Femtomaster FB 12, Zylux Corp., USA). Luciferase activities were normalized to renilla luciferase activity. The grouper larvae were sacrificed two days after DNA injection. The muscle tissue of injected region was collected and analysed its luciferase activity in the same manner as described above for cells. The data reported are the average of three replicate experiments.

### Production of recombinant MyoD protein

Full-length cDNA fragment encoding the orange-spotted grouper MyoD was cloned into the pET-29a vector to obtain the plasmid named pET29a-myoD and transformed into *E*. *coli* BL21 (DE3) cells for protein expression. Induction and purification of the recombinant protein was as described previously [47]. The concentration of the purified recombinant proteins was determined by using Pierce BCA Protein Assay Kit (Thermo Fisher Scientific Inc., USA). Bovine serum albumin (BSA) (Thermo Fisher Scientific Inc., USA) was used as the standard.

### Electrophoretic mobility shift assay (EMSA)

The oligonucleotide (Table 1) was labeled with fluorophore Cy3 and annealed with complementary oligonucleotide in annealing buffer (10 mM Tris-HCl pH 7.4, 10 mM MgCl_2_, 50 mM NaCl) by heating at 90°C for 5 min and allowing it to slowly cool to room temperature. The protein-DNA binding reaction was prepared by mixing the reaction buffer (20 mM HEPES pH 7.9, 50mM KCl, 5 mM MgCl_2_, 0.5 mM EDTA, pH8.0, 5 mM DTT, 5% glycerol) with the recombinant MyoD (2 μg) and poly [d(I-C)] (Sigma-Aldrich, USA) (0.1 ng) as the nonspecific competitor. The labeled probe was applied to the binding mixture, and the mixture was incubated for 30 min at room temperature. The total EMSA reactions were applied to a 6.5% nondenaturing polyacrylamide gel (0.5X TBE buffer, 6.5% acrylamide/bis-acrylamide (29:1), 6.5% glycerol, 0.1% ammonium persulfate, 0.1%TEMED). After electrophoresis at 100 V for 90 min, the gel was scanned by Biospectrum 500 (Ultra-Violet Products Ltd., USA).

### NNV infection

Grouper nervous necrosis virus (GNNV) was prepared as described in previous study [48]. Twenty-four hours post-transfection, the cells were washed with PBS and then infected with 10^4^ TCID_50_/mL of isolated GNNV in serum-free medium. After 1 hour of incubation, the cells were washed twice with PBS and then cultured in L-15 medium with 1% (v/v) FBS. Promoter activity was measured 24 hours post-infection.

### Data analysis and statistics

Statistical differences from at least three independent experiments were analyzed by ANOVA followed Tukey’s comparison. The standard error of mean was determined for each of the data sets of at least three independent experiments. A p-value < 0.05 was considered significant.
